# Conjuring up a ghost: structural and functional characterization of FhuF, a ferric siderophore reductase from *E. coli*

**DOI:** 10.1007/s00775-021-01854-y

**Published:** 2021-02-09

**Authors:** I. B. Trindade, G. Hernandez, E. Lebègue, F. Barrière, T. Cordeiro, M. Piccioli, R. O. Louro

**Affiliations:** 1 Instituto de Tecnologia Química e Biológica António Xavier (ITQB‑NOVA), Universidade Nova de Lisboa, Av. da República (EAN), 2780‑157 Oeiras, Portugal; 2 Université de Nantes, CNRS, CEISAM UMR 6230, 44000 Nantes, France; 3 Institut des Sciences Chimiques de Rennes-UMR 6226, Université Rennes, CNRS, 35000 Rennes, France; 4 Department of Chemistry, Magnetic Resonance Center (CERM), University of Florence, Via L. Sacconi 6, 50019 Sesto Fiorentino, Italy; 5 Consorzio Interuniversitario Risonanze Magnetiche di Metallo Proteine (CIRMMP), Via L. Sacconi 6, 50019 Sesto Fiorentino, Italy

**Keywords:** Ferric-siderophore reductase, Iron uptake, 2Fe–2S protein, Redox-Bohr effect

## Abstract

Iron is a fundamental element for virtually all forms of life. Despite its abundance, its bioavailability is limited, and thus, microbes developed siderophores, small molecules, which are synthesized inside the cell and then released outside for iron scavenging. Once inside the cell, iron removal does not occur spontaneously, instead this process is mediated by siderophore-interacting proteins (SIP) and/or by ferric-siderophore reductases (FSR). In the past two decades, representatives of the SIP subfamily have been structurally and biochemically characterized; however, the same was not achieved for the FSR subfamily. Here, we initiate the structural and functional characterization of FhuF, the first and only FSR ever isolated. FhuF is a globular monomeric protein mainly composed by α-helices sheltering internal cavities in a fold resembling the “palm” domain found in siderophore biosynthetic enzymes. Paramagnetic NMR spectroscopy revealed that the core of the cluster has electronic properties in line with those of previously characterized 2Fe–2S ferredoxins and differences appear to be confined to the coordination of Fe(III) in the reduced protein. In particular, the two cysteines coordinating this iron appear to have substantially different bond strengths. In similarity with the proteins from the SIP subfamily, FhuF binds both the iron-loaded and the apo forms of ferrichrome in the micromolar range and cyclic voltammetry reveals the presence of redox-Bohr effect, which broadens the range of ferric-siderophore substrates that can be thermodynamically accessible for reduction. This study suggests that despite the structural differences between FSR and SIP proteins, mechanistic similarities exist between the two classes of proteins.

**Supplementary Information:**

The online version contains supplementary material available at 10.1007/s00775-021-01854-y.

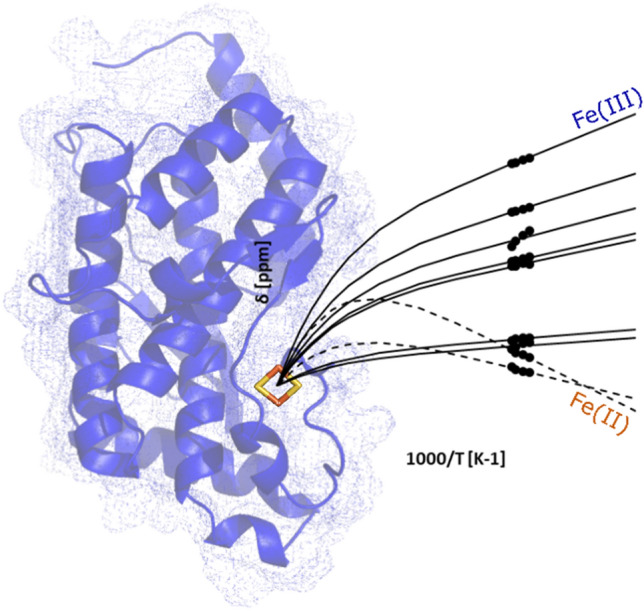

## Introduction

“Structure without function is a corpse; function without structure is a ghost”

(Vogel and Wainwright, 1969)

The Great Oxidation Event made the hitherto abundant iron a trace element as a consequence of precipitation of iron oxides [1]. Iron has, nonetheless, remained an essential element for nearly all organisms [2, 3]. To overcome iron limitation, almost all known bacterial species use siderophores, small molecules that scavenge iron from the extracellular environment, forming Fe(III)-complexes which are then taken up inside the cell [4, 5]. Different TonB-dependent receptors recognize different siderophores into the periplasm and their transport across the cytoplasmic membrane is dependent on ABC transporters. Once inside the cytoplasm, iron can be released via the action of esterases or via reduction by ferric-siderophore reductases [5]. The laboratory strain *Escherichia coli* K-12 can use diverse siderophores including hydroxamates (e.g. ferrichrome, ferrioxamine B, coprogen) and catecholates (e.g. enterobactin, yersiniabactin, salmochelin) and it contains at least five different uptake systems, including the ferrichrome operon (fhuACDB) and the enterobactin uptake system (fepA, fepB, fes and fepCDG genes) [6–10]. In the cytoplasm, two distinct ferric-siderophore reductases have been isolated. One is YqjH that belongs to the SIP (Siderophore-Interacting Protein) subfamily, which is able to catalyze the release of iron from Fe(III)-triscatecholates and Fe(III)-dicitrate. The other is FhuF, from the FSR (Ferric-Siderophore Reductase) subfamily that showed specificity for a group of hydroxamate-type siderophores, since iron removal from coprogen, ferrichrome, and ferrioxamine B is significantly reduced in FhuF-defective mutants [8, 11, 12]. Very little is known regarding the structure and function of the FSR subfamily of proteins, given the instability of the pure proteins [11]. FhuF is the only protein of the FSR subfamily ever isolated. Its transcription is derepressed by low iron levels via the iron regulator Fur, and repressed by OxyR, an oxidative response regulator [10]. This protein contains a 2Fe–2S cluster with unusual properties, including the unprecedented binding motif sequence C–C–*x*_10_–C–*x*_2_–C, unusual EPR *g*_*z*_ value for this kind of cluster (*g*_*z*_ = 1.994), and unusual Mössbauer parameters with a low quadrupole splitting in the oxidized form (∆EQ,4.2 K = 0.474 mm s^−1^) and unusually high quadrupole splitting for the Fe(III) component of the reduced form (∆EQ190 K = 0.978 mm s^−1^) [8, 11]. Furthermore, previous studies show that FhuF is truncated at the N-terminal end, and that it is loosely associated with the cytoplasmic membrane, since it is possible to purify FhuF from both cytoplasmic and membrane fractions [11]. Here, we used a combination of Circular Dichroism (CD), SAXS, Rosetta modelling, electrochemistry and paramagnetic NMR spectroscopy to advance the structural and functional characterization of FhuF from *E. coli* K-12.

## Materials and methods

### Protein production and purification

The plasmid pKF191, derived from pET-19b that codes for His-tagged FhuF protein was isolated and transformed into BL21 DE3 competent cells for expression [8, 11]. Freshly transformed cells were grown in Terrific Broth medium supplemented with 100 mg L^−1^ ampicillin at 37 ºC, 160 rpm until they reached an OD of 0.7, the temperature was then decreased to 30 ºC and cells were collected after 4 h. Cells were harvested by centrifugation for 10 min at 11,305*g* and were then cooled to – 20 ºC. The cells were later defrosted and resuspended in 20 mM Potassium Phosphate buffer pH 7.6, 300 mM NaCl with a protease-inhibitor cocktail (Roche) and DNase I (Sigma) prior to a three-pass cell disruption at 6.9 MPa using a French press. The lysate was ultracentrifuged at 204,709*g* for 90 min at 4 ºC to remove cell membranes and debris. FhuF was purified from the supernatant using a His-trap affinity column (GE Healthcare) using a stepwise elution method. The fraction containing FhuF eluted at 20 mM Potassium Phosphate pH 7.6, 300 mM NaCl and 250 mM imidazole. Eluted fractions were analyzed by SDS-PAGE with Blue-Safe staining (NZYTech) and UV–visible spectroscopy to select fractions containing pure FhuF. The imidazole was removed and FhuF was concentrated at 36 ºC using an Amicon^®^ Ultra Centrifugal Filter with a cutoff of 10 kDa. For the SAXS data collection, FhuF fractions were further purified using a Superdex 75 10/300 GL from GE Healthcare at 1 mL min^−1^. Samples were kept at 30 ºC with 0.5 mM of sodium azide and aliquots were sent for N-terminus sequencing to confirm the identity of the purified protein.

### Circular dichroism

The Far-UV CD spectra of a 6.1 µM FhuF sample in 20 mM Potassium Phosphate pH 7.6 was recorded on a Jasco-815 spectrophotometer using a 1 mm quartz cell for high performance (QS) (Hellma Analytics). All CD measurements are an average of four accumulations collected in the 190–260 nm wavelength range using a 0.1 nm data pitch, and 2 nm bandwidth at 50 nm/min. The CD spectra were input into the BeStSel webserver to predict secondary elements content [13]. The thermal denaturation of FhuF was followed by monitoring changes in spectral features as a function of temperature ranging from 4 to 81 ºC with 7 ºC steps. The values for the unfolded fraction ${f}_{\mathrm{U}}$ were obtained by linear extrapolation of the folded ${\theta }_{\mathrm{F}}$ and unfolded ${\theta }_{\mathrm{U}}$ baselines into the transition zone using the following equation:

1 $${f}_{\mathrm{U}}=\frac{\theta -{\theta }_{\mathrm{F}}}{{\theta }_{\mathrm{U}}-{\theta }_{\mathrm{F}}}$$

where $\theta$ is the mean residue ellipticity. By fitting to a sigmoidal equation, we extracted the melting temperature (*T*_m_) of FhuF (i.e., the temperature when ${f}_{\mathrm{U}}$ = 0.5).

### Small-angle X-ray scattering

Synchrotron SEC-SAXS data on FhuF was collected on the B21 (ESRF, Grenoble, France) beamline exploiting its *in-line* HPLC system (Agilent 1200 HPLC). To this end, we injected 50 μL samples with 8.9 mg mL^−1^ of SEC purified protein in a 4.6 mL Shodex KW402.5-4F size exclusion column at a flow rate of 0.16 mL min^−1^. Two-second frames were acquired using a Pilatus 2 M pixel detector. Data collection conditions are described in Table SI, and no measurable radiation damage or significant signs of interparticle interference or aggregation were detected. The SEC mobile phase consisted of 20 mM potassium phosphate buffer pH 7.6, 150 mM NaCl. The scattering intensities from the respective monomeric elution single-peak region were integrated and buffer subtracted to produce the SAXS-profile of FhuF using the ScÅtter software [14]. Further processing was performed using the *ATSAS* software suite [15]. The *P(r)* distribution function was obtained by indirect Fourier Transform. The *R*_g_ value was estimated by applying the Guinier approximation in the range *s* < 1.3/*R*_g_. The SEC-SAXS profile raw data were deposited in the repository for small-angle scattering data SASBDB under the project "SAXS of FhuF—A ferric-siderophore reductase" with the accession code SASDJ28 [16]. From SEC-SAXS data, a low-resolution ab initio molecular envelope was generated for FhuF, with the program DAMMIF using the *ATSAS* package using the pair-wise distance distribution (*P(r)*) calculated from range 0.012 < *s* < 0.37 Å^−1^ [15]. Twenty independent models were generated, and then superimposed and averaged to define the most populated volume and test the robustness of the models.

### FhuF modeling

The model for FhuF was built by homology modeling using as templates the known crystal structures of proteins bearing the FhuF domain, including enzymes involved in iron siderophore biosynthesis in pathogenic bacteria [17]. There are 36 instances of this domain found in the PDB database. The multi-template modeling was performed with RosettaCM using evolutionary coupling-derived distance restraints [18, 19]. With RosettaCM, the most homologous portions from the multiple templates are hybridized into a single model while modeling the missing residues de novo. We sampled the diversity of conformational space by building a total of 4000 models, denoted pool. We scored each model by its Rosetta energy score (*Ei*) and relative agreement to the SEC-SAXS data (reduced ${\chi }_{i}^{2}$) using the following hybrid-scoring function:

2 $${Z}_{i}={Z}_{\mathrm{SAXS}}^{i}+{Z}_{\mathrm{Rosetta}}^{i}={w}_{\mathrm{saxs}}\bullet \left(\frac{{\chi }_{i}^{2}-\langle {\chi }^{2}\rangle }{{\sigma }_{\mathrm{SAXS}}}\right)+\left(1-{w}_{\mathrm{saxs}}\right)\bullet \left(\frac{{E}_{i}-\langle E\rangle }{{\sigma }_{\mathrm{Rosetta}}}\right)$$

where ${\chi }_{i}^{2}$ and ${E}_{i}$ were standardized to ${Z}_{\mathrm{SAXS}}^{i}$ and ${Z}_{\mathrm{Rosetta}}^{i}$, respectively, using the mean values ($\langle {\chi }^{2}\rangle$ and $\langle E\rangle$) and standard deviations (${\sigma }_{\mathrm{SAXS}}$ and ${\sigma }_{\mathrm{Rosetta}}$) of the pool, with ${w}_{\mathrm{saxs}}$ defining the weight of each term (Borges et al., 2020). The reduced ${\chi }_{i}^{2}$ was given by CRYSOL 3.0 [15].

### NMR spectroscopy

#### ^1^H temperature dependence experiments

A sample of approximately 500 μM of oxidized FhuF in 20 mM Potassium Phosphate buffer pH 7.6 with 300 mM NaCl was lyophilized and solubilized in D_2_O (99.9 atom %) for ^1^H temperature dependence experiments. Reduced FhuF was obtained using the same sample by degassing it and by adding an excess of sodium borohydride in an anaerobic chamber. ^1^H NMR experiments were performed on a Bruker Avance II 500 MHz NMR spectrometer equipped with a 5 mm BBI probe. A total of 61,440 transients were acquired using the super-WEFT pulse sequence (180-τ-90-AQ) with 103 ms of recycle time and τ values of 45 ms, to dampen the diamagnetic signals and suppress the solvent.

For each temperature, in degrees Celsius, the chemical shift of each proton signal [(Av/vo)^con^_*j*_] was referenced to TMS at 0 ppm using the H_2_O signal as a secondary reference (5.11–0.012 × *T* ppm). The temperature dependence of the contact shift of the cysteine protons was determined according to the Van Vleck formalism:

3 $${\left(\frac{\Delta v}{{v}_{0}}\right)}_{j}^{\mathrm{con}}=\frac{2\pi g{\mu }_{\mathrm{B}}}{3{\gamma }_{I}kT}.\frac{{A}_{j}}{h}.{\sum }_{i}\frac{{C}_{ji}{{S}^{^{\prime}}}_{i}\left({{S}^{^{\prime}}}_{i}+1\right).\left(2{{S}^{^{\prime}}}_{i}+1\right)\mathrm{exp}\left(\frac{-{E}_{i}}{kT}\right)}{\left(2{{S}^{^{\prime}}}_{i}+1\right)\mathrm{exp}\left(\frac{-{E}_{i}}{kT}\right)}$$

where *g* is the Free-spin electron g factor, *μ*_B_ is the Bohr magneton, *γ*_I_ is the free-electron magnetogyric ratio for the ^1^H, *k* is the Boltzmann constant, *T* is the absolute temperature, *A*_*j*_ is the electron-nuclear coupling constant, *h* is the Planck constant, *C*_*ji*_ reflect the contribution of each spin (*S*_1_ and *S*_2_) to the total spin (*S*’) for each *i* level [20–23]. The *E*_*i*_ are the energy values of the eigenstates *S*’ ranging from |*S*_1_–*S*_2_| to |*S*_1_ + *S*_2_| described by the perturbative Heisenberg Hamiltonian as a function of the magnetic exchange coupling constant *J* according to Eq. (4):

4 $$E_i = \frac{1}{2}JS_i^{\prime} \left( {S_i^{\prime} + 1} \right)$$

Equation (3) was fit to the experimental data using the solver routine in MSExcel using standard parameters. Standard errors were determined from the diagonal elements of the covariance matrix considering 1 ppm experimental uncertainty in the chemical shift measurements [24].

### ^15^N^13^C FhuF: binding experiments

Experiments were performed at 305 K using a Bruker AVANCE III spectrometer operating at 800 MHz equipped with a TCI cryoprobe. The reference ^1^H-^15^N TROSY-HSQC experiment was acquired with 64 transients using the pulse sequence trosyetf3gpsi from the BRUKER catalogue with 250 μM of ^15^N-^13^C labeled FhuF in 20 mM Potassium Phosphate buffer pH 7.0 with 300 mM NaCl. The TROSY version of the HSQC was chosen, because it provided sharper signals that facilitated the analysis. Binding experiments were performed using samples of 200 μM of ^15^N-^13^C labeled FhuF against increasing amounts of ligand (L): ferrichrome, apo-ferrichrome. All are oxidized to prevent electron transfer upon binding. Following each addition a 16 transients ^1^H-^15^N TROSY-HSQC experiment was recorded. Chemical shift perturbations (Δ_bind_) of the NMR signals from FhuF (Protein, P) were plotted against the molar ratio (*R*) of [L]/[P]. The data were fitted using least-squares minimization to a 1:1 binding model using equations [25]:

5 $${\Delta \delta }_{\mathrm{bind}}=\frac{1}{2}{\Delta \delta }_{\mathrm{bind}}^{\infty }\left(A-\sqrt{\left({A}^{2}-4R\right)}\right)$$

6 $$A =1+R+\frac{Kd\left({\left[P\right]}_{0}R+{[L]}_{0}\right)}{{\left[P\right]}_{0}{[L]}_{0}}$$

where ${\Delta \delta }_{\mathrm{bind}}^{\infty }$ is the maximal chemical shift perturbation of the NMR signals resulting from the complex formation between the protein and the ligand, and [*P*]_0_ is the initial protein concentration and [*L*]_0_ is the stock concentration of ligand. Only chemical shift perturbations (${\Delta \delta }_{\mathrm{bind}}$) equal to or larger than 0.025 ppm were considered significant. The standard deviation of the fitted value of *K*_*d*_ was calculated using all data.

### Electrochemical experiments

Cyclic voltammetry was performed in a three-electrode cell with an edge plane pyrolytic graphite disk electrode (PGE, 3 mm diameter) obtained from IJ Cambria Scientific Ltd. as the working electrode. All cyclic voltammetry measurements were recorded using an Ag/AgCl, KCl 3 M as reference electrode and a graphite rod as counter electrode. Electrochemical experiments were performed at room temperature with a SP-300 potentiostat driven by EC-Lab V10.40 software from Bio-Logic (Bio-Logic Science Instruments, France). PGE was immersed in diluted protein solution (100 µM) for 3 days before electrochemical measurements in protein-free aqueous electrolyte (20 mM potassium phosphate buffer) at different pH values between pH 5 and pH 9. All solutions were degassed by bubbling argon for 10 min before each measurement. To facilitate the reading, the potentials discussed in the manuscript are reported in V vs SHE (E/V vs SHE = E/V vs Ag/AgCl + 0.210 V).

## Results

### FhuF is a globular helix-bundle protein

**Fig. 1:**
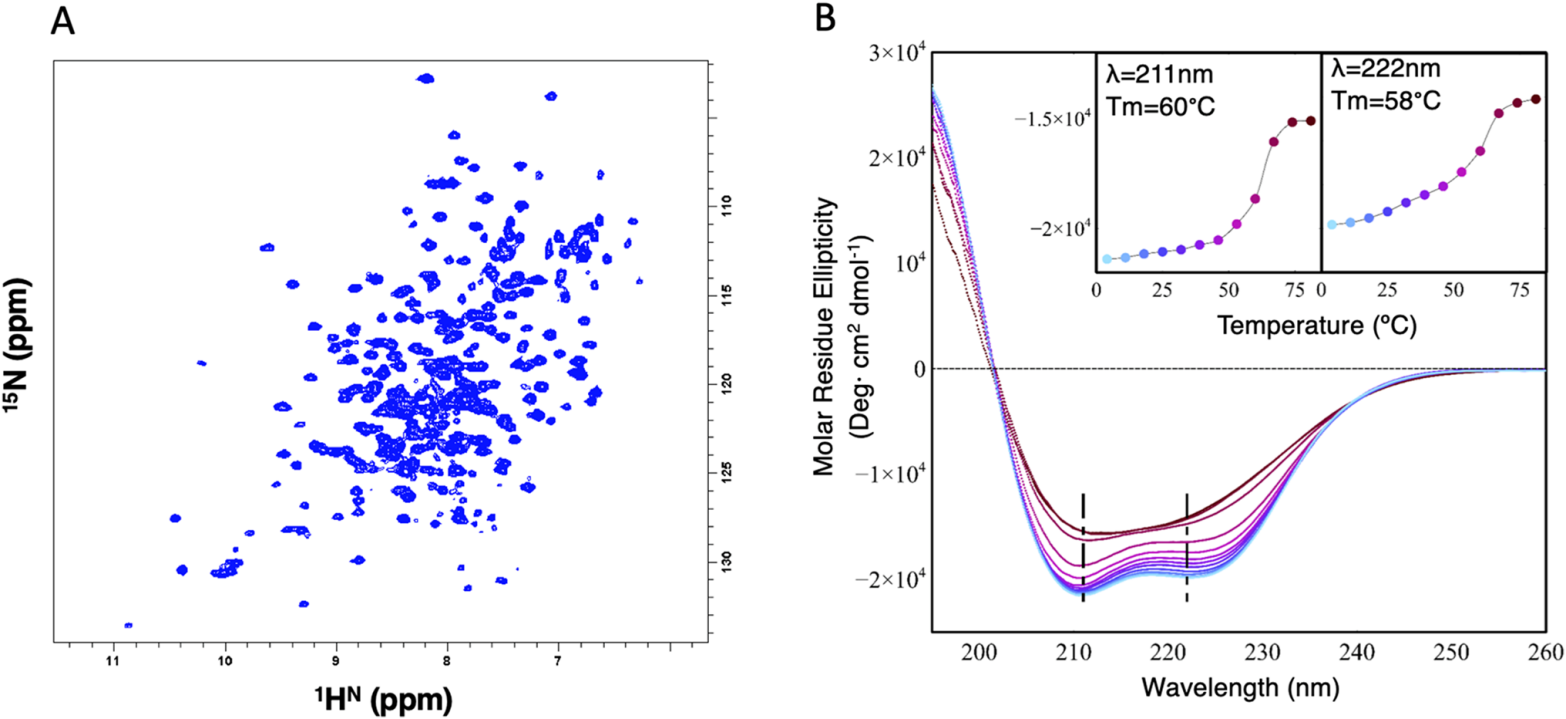
FhuF is a folded protein. **a** 2D ^1^H^15^N TROSY-HSQC spectrum of ^15^N/^13^C-labeled FhuF collected at 305 K on a Bruker Avance III 800 MHz spectrometer. The resonance map has the typical fingerprint of a folded protein with large chemical shift dispersion. **b** CD spectra of FhuF at different temperatures ranging from 4 and 81 ºC with successive 7 ºC intervals. (Inset) Thermal denaturation as measured by changes in the signal at 211 and 222 nm

Expression and purification of FhuF gave rise to a folded and soluble functional domain encompassing residues 18–262. The protein eluted from the SEC column at a volume corresponding to an apparent molar mass of ca 26.9 kDa, demonstrating that FhuF (28 kDa) is monomeric, migrating through the column as a folded protein. NMR data in solution revealed that FhuF is indeed well-folded at 32 ºC (Fig. 1a) as seen from the broad resonance dispersion of the ^1^H–^15^N fingerprint, reflecting the presence of secondary and tertiary structure elements, thus compatible with well-folded protein. The CD profile of FhuF also indicates the presence of stable structural elements within the overall protein structure (Fig. 1b), displaying positive values below 200 nm and two negative bands at 208 and 222 nm, commonly associated with α-helical conformations. BeStSel predicts a helical content of 55% and 40% of unordered/turn elements with a small contribution of antiparallel β-sheet [13]. The thermal denaturation curve of FhuF followed by the changes in the CD signals at 211 and 222 nm show a cooperative folding-unfolding transition with a Tm of 58–60 ºC, indicating that the protein has a defined tertiary structure (Fig. 1b, inset) that unfolds by increasing temperature.

**Fig. 2:**
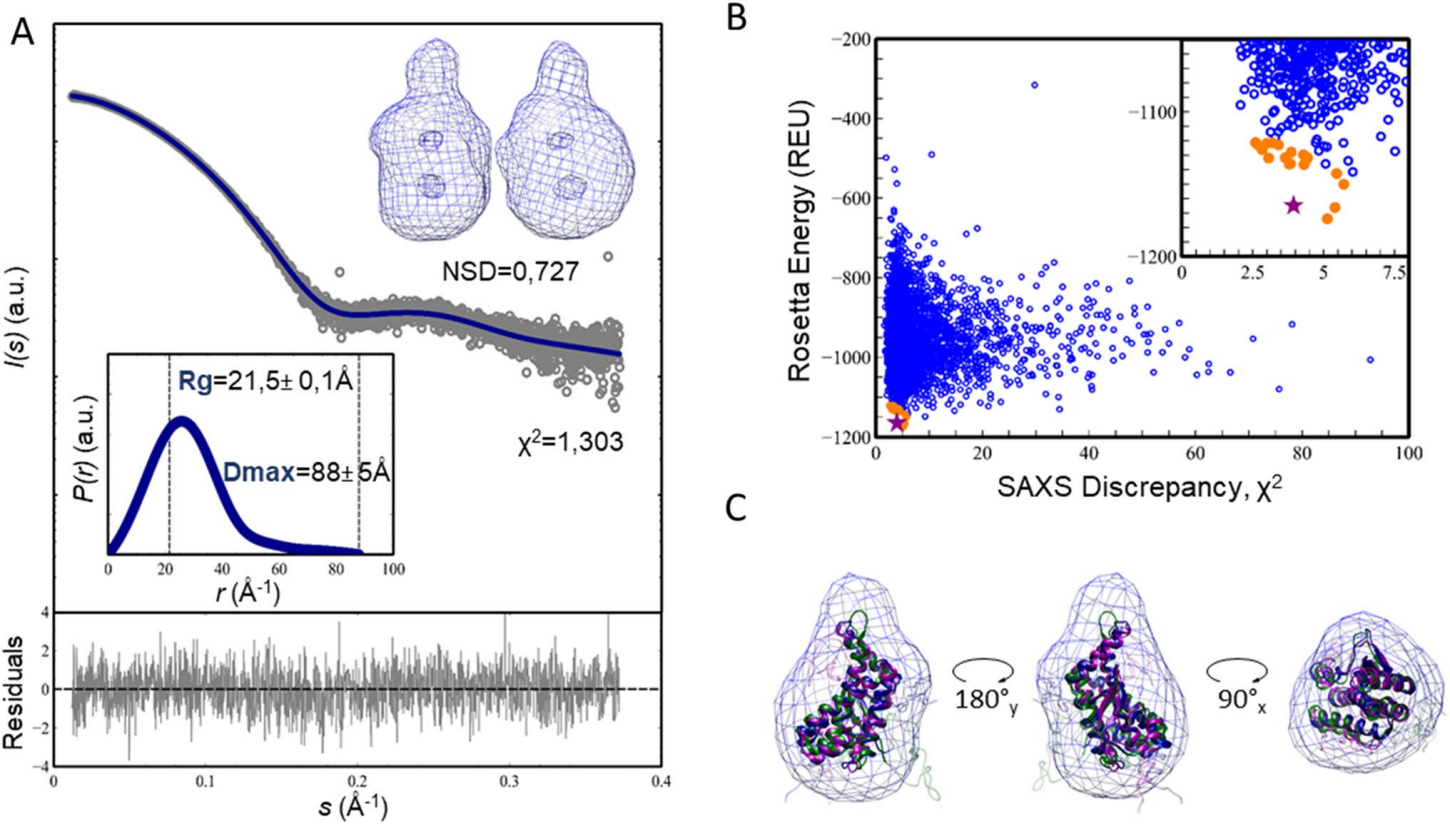
Structural model of FhuF. **a** SAXS intensity of SEC-purified FhuF (gray circles), *I*(s), is represented in logarithmic scale as a function of the momentum of transfer, s. The dark blue line corresponds to the scattering profile calculated from the ab initio model that best fitted the experimental data (*χ*^2^ = 1.3). Point-by-point deviations of the fitting are in the bottom panel. The top inset shows the SAXS-generated ab initio envelope obtained by clustering and averaging 20 independent models, with a Normalized Spatial Discrepancy (NSD) of 0.727 Å. The bottom inset shows the pair-wise distance distribution (*P*(*r*)) of FhuF. The derived Rg and Dmax values are displayed in dashed lines. **b** Rosetta energy vs. SAXS discrepancy, *χ*^2^ scatter plot for the 4000 structure-predictions of FhuF. REU stands for Rosetta Energy Units. Inset shows expanded scale to highlight the 20 models with the lowest scoring in orange dots, and the best-model as a purple star. **c** SAXS ab initio reconstruction of FhuF (blue envelope) containing the three best-scored homology models. The regions with high uncertainty are transparent

We have employed SAXS to probe further the overall structure of FhuF. Our synchrotron SEC-SAXS data confirmed that FhuF is also a monomeric globular particle in solution, with a radius of gyration of 21.5 ± 0.10 Å and a maximum distance of 88.0 ± 5 Å (Table 1) (Fig. 2a).

**Table 1:** SEC-SAXS data analysis

	FhuF
Overall parameters	
* R*_g_ (Å) [from *P(r)*]	21.49 ± 0.10
* R*_g_ (Å) [from Guinier]	21.51 ± 0.10
* D*_max_ (Å)	88.0 ± 5.0
Porod volume estimate, *V*p (Å^3^)	37,902.8
Molecular weight estimate (kDa)^a^	25.8 (7.8%)
Oligomeric state	Monomer
Software	
SEC-SAXS data integration	ScÅtter
* P(r)*	GNOM 5.0
Ab initio modelling/ < NSD > (Å)^b^	DAMMIF^c^/0.727 ± 0.049 Å
Simulated SAXS	CRYSOL
SASBDB accession code	SASDJ28

^a^Calculated with SAXSMoW 2.1[26]. The discrepancy to the sequence weight of FhuF∆1–17 is given inside parentheses

^b^Mean ± std

^c^Refinement with DAMMIF [15]

**Fig. 3:**
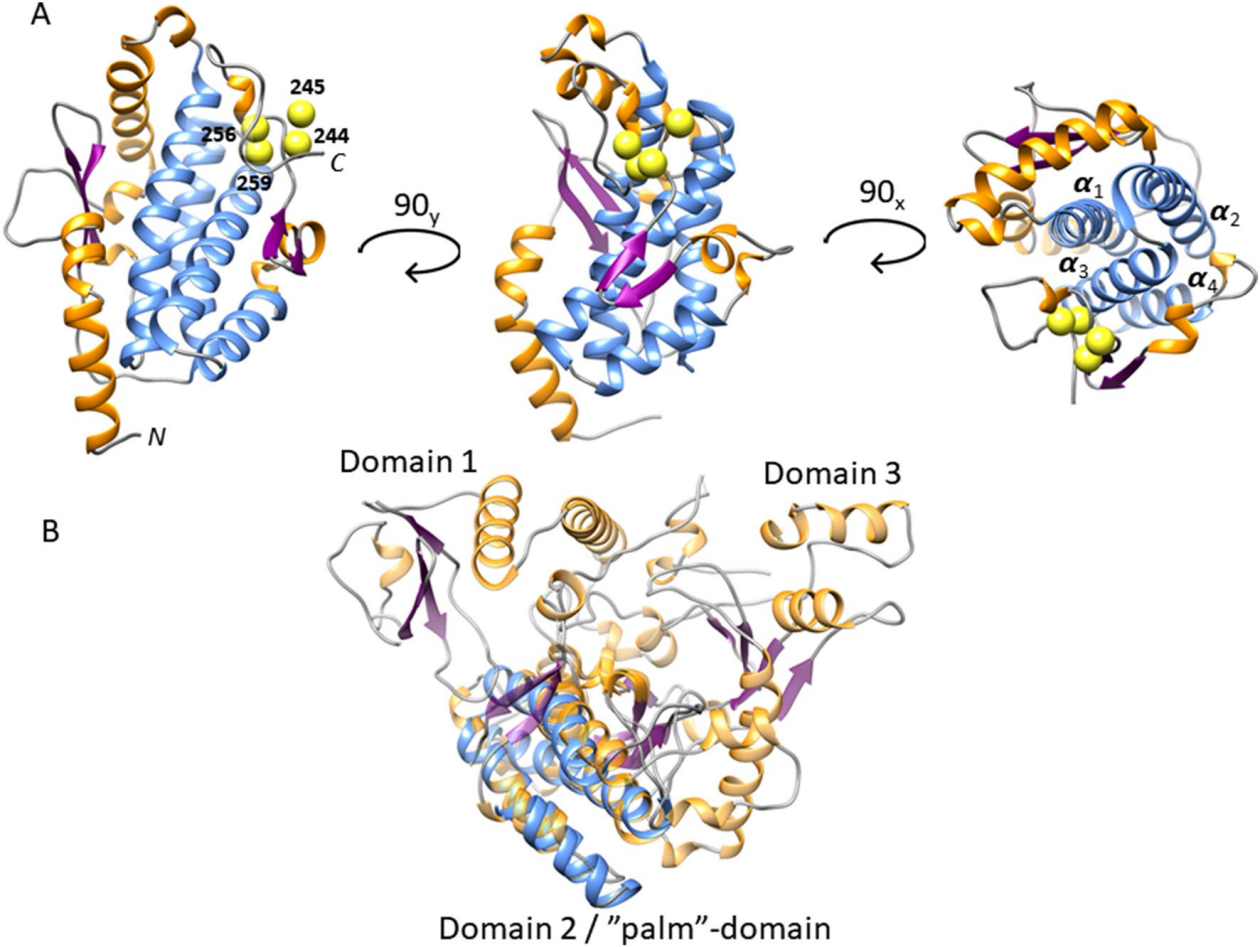
Relation of overall FhuF structural model with its closest homologues. **a** Ribbon representation of the best-scored model. The core four-helix bundle is colored in light blue, other helices in orange, and beta-sheets in purple. The SG atoms of the cysteines known to coordinate the 2Fe–2S centre at the C-terminal are depicted as yellow spheres. **b** Overlay of FhuF model and AcsD X-ray structure (PDB:2W02) in transparent ribbon representation

The Kratky representation of the protein SAXS data (Figure S1) is bell-shaped, as expected for highly spherical/globular proteins, contrary to disordered proteins that do not display a clear maximum [27]. Nevertheless, the smooth asymmetrical tailing in its pairwise distance distribution (*P(r)*) suggests the presence of moderate flexibility or a slight deviation from a compact sphere (Fig. 2a). Accordingly, the SAXS-derived low-resolution structure is oblong with a spatially separated lobe and two regions inside the envelope with lower density probability resembling small cavities. To gain more structural insight into FhuF, we used Rosetta to predict its potential structure, using multiple templates sharing sequence similarity with FhuF, and incorporating evolutionary coupling constraints into the homology modeling protocol [18, 19]. Interestingly, all identified templates were enzymes involved in the iron siderophore biosynthesis process with a FhuF-like domain within their overall structure. Examples are the AcsD from the plant pathogen *Pectobacterium chrysanthemi* and IucA from the human pathotype *Klebsiella pneumonia* [17, 28]*.* Both have three domains that resemble a cupped hand and these are designated thumb domain 1, palm domain 2, and fingers domain 3. The palm domain is FhuF-like, and in the context of AcsD/IucA, contributes with the active site residues. We only used the palm-domain regions as templates. To improve the modeling and eliminate false-positives, we used SAXS to score, discriminate, and validate all FhuF models. SAXS-based approaches were successfully applied in loop modeling, distinguishing protein–protein interfaces as well as improving structure prediction accuracy from unbiased MD simulations [29–32]. Herein, we used SAXS information combined with Rosetta energy to identify those models with the lowest possible scoring energy, and which best fit the SAXS data [29]. Figure 2b shows the energies of all models with respect to their SAXS discrepancy scores (reduced χ2). The best-scored models display a similar well-folded central core, faithfully matching the ab initio envelope's high-density region (Fig. 2c). The C-terminal part containing the four conserved cysteine residues from the 2Fe–2S cluster is less defined in the models, mostly due to the high uncertainty in the iron-cluster geometry and lack of structural constraints driving the modeling. FhuF contains a 2Fe–2S cluster with unprecedented binding motif sequence Cys–Cys–*x*_10_–Cys–*x*_2_–Cys. Without a template for the iron-cluster, predicting this region de novo remains a challenging task in structural biology, even with the incorporation of evolutionary coupling constraints [19]. Nevertheless, in most models, the cysteines are spatially clustered, at a distance adequate to accommodate a 2Fe–2S cluster (Fig. 3a). The well-defined core of the model is primarily α-helical, in agreement with the CD data, comprising a four-helix bundle (α1:88–114; α2:153–177; α3:180–202; α4:205–216) sandwiched between a three-stranded antiparallel β-sheet (β1:53–56; β2:125–129; β3:135–140) and a two-stranded antiparallel β-sheet. The overall fold is analogous to the palm domain within AcsD/IcuA-like proteins (Fig. 3b).

### Paramagnetic NMR suggests that the core of the 2Fe2S cluster structure is similar to that of other ferredoxins

**Fig. 4:**
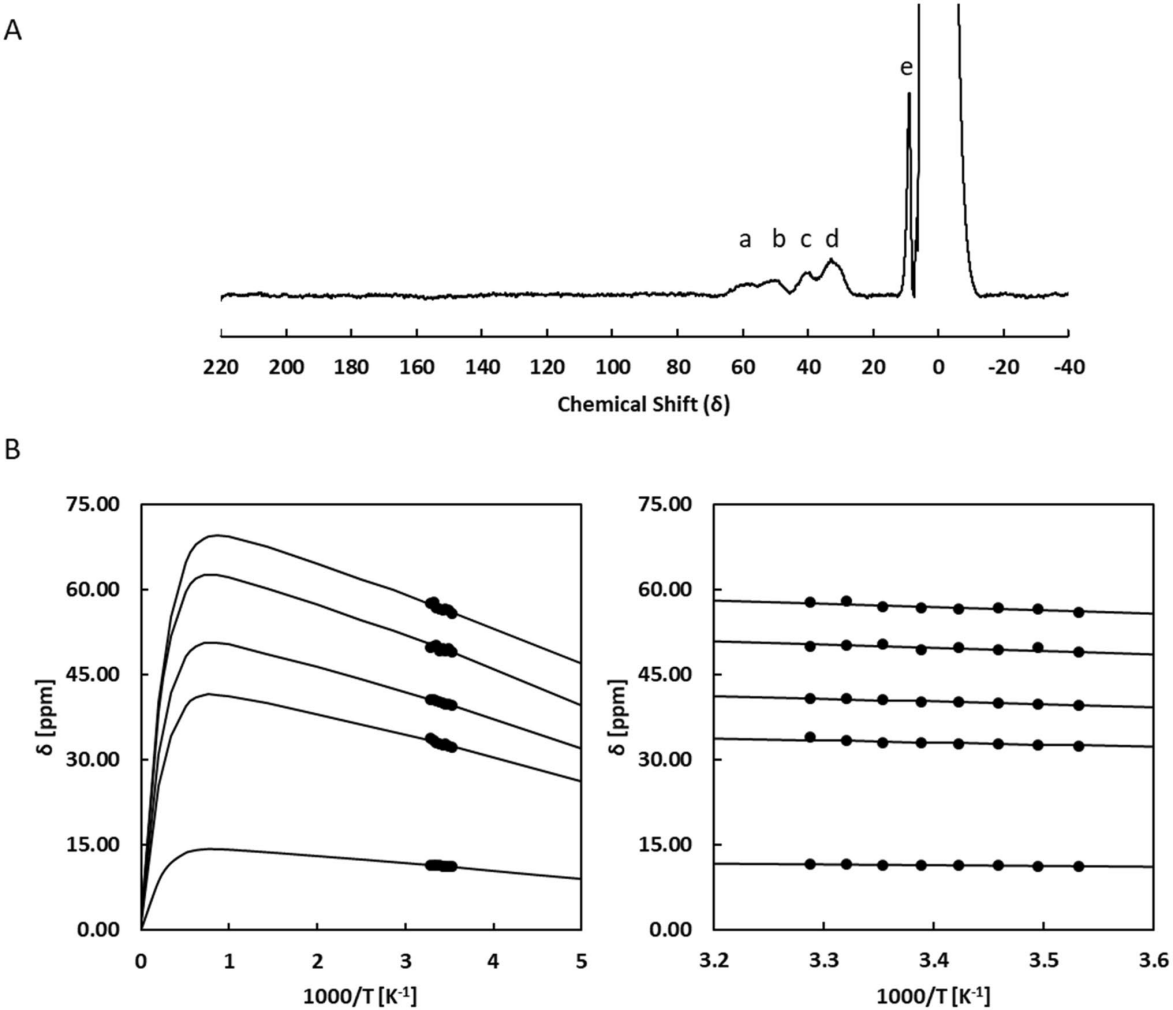
Oxidised FhuF has a thermally populated paramagnetic excited state. 1D ^1^H NMR spectra of oxidized FhuF **(a)**. Temperature dependence of hyperfine shift of β-CH_2_ and α-CH protons of oxidized FhuF **(b)**. Lines represent the fitting of Eq. (3) to the experimental data, with Ai/h ranging from 3.07 MHz to 0.63 MHz using a single J value of 300 cm^−1^ with a standard error of 2.5 cm^−1^

**Fig. 5:**
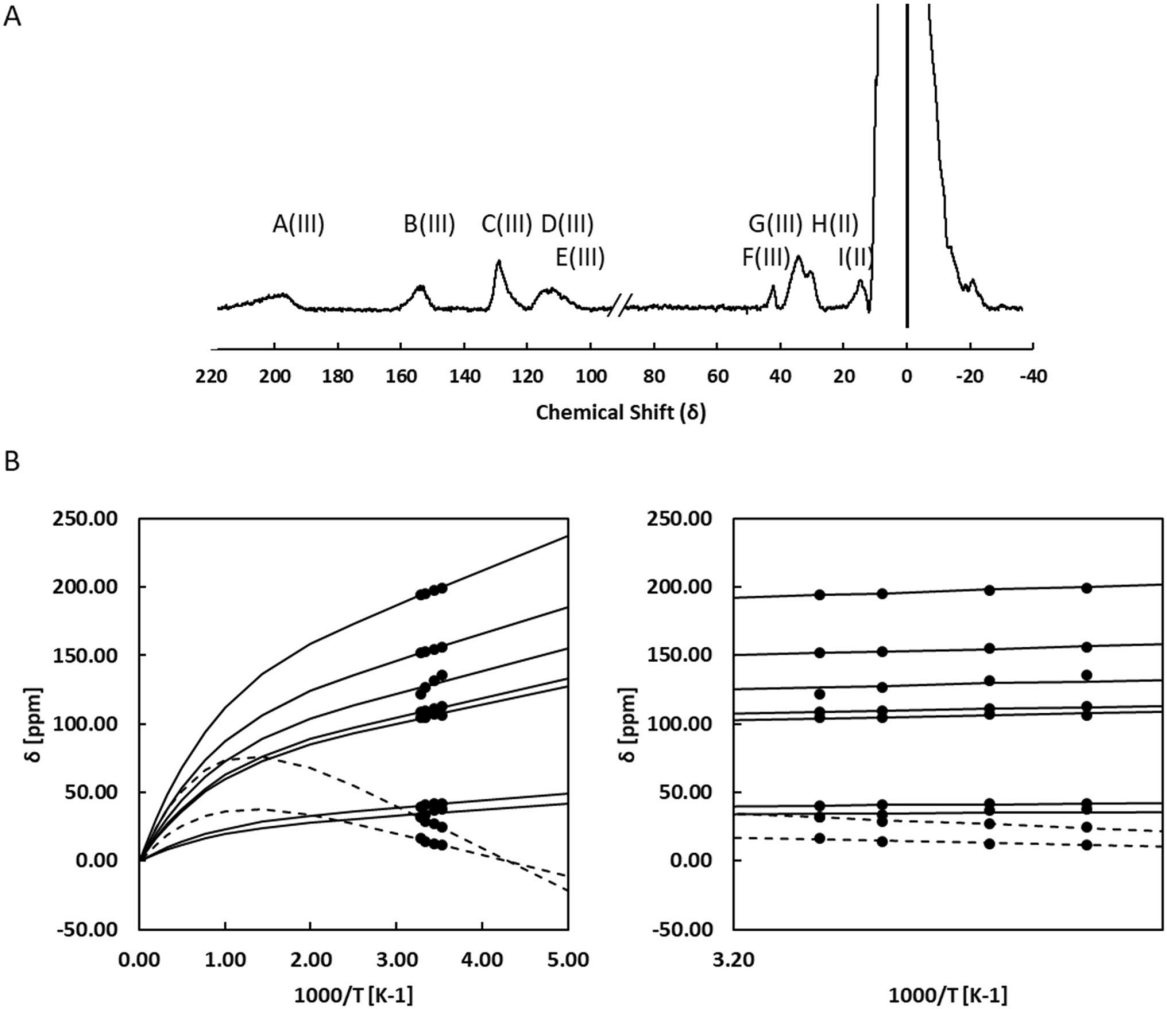
Reduced FhuF localizes the electron in a specific iron. 1D ^1^H NMR spectra of reduced FhuF **(a**). Temperature dependence of hyperfine shift of β-CH_2_ and α-CH protons of reduced FhuF **(b)**. Lines represent the fitting of Eq. 3 to the experimental data, with Ai/h ranging from 2.30 MHz to 0.31 MHz and using a single J value of 115 cm^−1^ with a standard error of 2.5 cm^−1^

The unprecedented binding motif of the 2Fe2S cluster, involving cysteines 244, 245, 256 and 259 together with the unusual g_z_ value reported for the reduced form and the unusual Mössbauer parameters for the ferric iron of the reduced form of the cluster suggested the presence of a novel 2Fe–2S cluster structure [8, 11]. This is expected to affect the electronic structure parameters of the cluster that can be explored by NMR spectroscopy probing the paramagnetic effect on the protons of the cysteines coordinating the cluster. Indeed, there are paramagnetically shifted signals both in the oxidized and reduced forms of FhuF. In the reduced form these shifts arise from the presence of an unpaired electron in the 2Fe–2S cluster. In the oxidized form these arise from the presence of thermally populated paramagnetic excited states at room temperature even though the ground state of the cluster is diamagnetic. Figures 4 and 5 show that FhuF is amenable to detailed characterization of the electronic properties of the 2Fe2S cluster by paramagnetic NMR spectroscopy, because it displays resolved features in both oxidation states. FhuF is unusual in this respect, because vertebrate 2Fe2S ferredoxins typically display unsuitable spectra in the reduced state, whereas the opposite is observed for 2Fe2S plant ferredoxins [33]. Figure 4a shows the downfield region of 1D ^1^H NMR spectra of oxidized FhuF. The spectrum of FhuF has a similar pattern of signals to that observed in HuFd (Human ferredoxin), where a total of five broad peaks (a–e) are observed, one at 11 ppm (e), and four between 32 and 57 ppm (a–d) [33]. All five signals exhibit anti-Curie temperature dependence (Fig. 4b), i.e., signals shift further downfield as the temperature is increased, consistent with an antiferromagnetically coupled Fe(III)–Fe(III) pair, as reported by EPR and Mössbauer spectroscopy [11]. A 2Fe–2S cluster containing two antiferromagnetically coupled Fe(III) has an *S* = 0 ground-state which is diamagnetic, thus the paramagnetic effect observed arises from thermal population of excited spin-states in the orbital manifold. The temperature dependence of these signals is well reproduced by the VanVleck formalism using the *E*_i_ values obtained with *J* = 300 cm^−1^ and *A*_*j*_*/h* ranging between 0.63 and 3.07 MHz. This *J* value is in the high range compared with those reported for spinach and algal ferredoxins (*J* = 290 cm^−1^ and 185 cm^−1^) based on measurements of the temperature dependence of paramagnetic shifts of cysteine ligands to the clusters, the temperature dependence of magnetic susceptibility and on ENDOR, Mössbauer and EPR data [34–36]. The *A*_j_*/h* values for β-CH_2_ cysteine protons fall in the expected range of 1 to 3 MHz obtained from proton ENDOR data on a [Fe_4_S_4_]^3+^ and rubredoxin models [21, 37]. The lower value of 0.63 MHz, calculated for peak e, therefore, also argues for assigning this signal to an α-CH proton of a cysteine bound to the cluster [21]. Therefore, by analogy with HuFd, the peak at 11 ppm most likely accounts for an α-hydrogen of one of the four ligated cysteines, and the peaks between 57 and 32 ppm arise from the cysteinyl β-hydrogens [33].

The 1D ^1^H NMR spectrum of reduced FhuF (Fig. 5a) revealed nine peaks, five (A(III)–E(III)) between 199–104 ppm and four between 42 and 11 ppm (F(III)–I(II)). Unlike the oxidized spectrum, the reduced spectrum of FhuF does not resemble the spectra of reduced HuFd. Instead it resembles the reduced spectrum of Anabaena 7120 vegetative ferredoxin (Vfd) [38]. Despite the similar pattern, the signals of the Anabaena ferredoxin do not reach beyond 140 ppm, whereas in Fhuf, the most downfield signals reach to nearly 200 ppm. This suggests that one of the iron coordinating cysteines has an unusually strong bond. Of the nine peaks, the two less downfield-shifted signals exhibit anti-Curie temperature dependence (H(II)-I(II)) while the remaining seven, more far-shifted, exhibit Curie temperature dependence (A(III)-G(III)) (Fig. 5b). Signals with anti-Curie temperature dependence are assigned to protons of cysteines that are ligated to Fe(II), whereas signals with Curie temperature dependence are assigned to protons of cysteines that are ligated to Fe(III) [21, 33]. The observation of seven peaks with Curie temperature dependence and the differences in intensities of some peaks, for example peak F appears to be of lower intensity, argue for the presence of heterogeneity in the cluster environment in the reduced Fhuf. Indeed, the observation of one clear extra peak from the 6 expected for a pair of βCH_2_ and one αCH for each cysteine suggest that in the reduced state there are at least two coordination modes for the Fe (III). Nonetheless, Fig. 5b shows that the experimental data are well reproduced using *J* = 115 cm^−1^ and *A*_*j*_*/h* values between 0.31 and 2.13 MHz. Only peak C shows an apparent temperature dependence that would require distinct and unrealistically high values for *A*_*j*_*/h* and *J*. Nonetheless, the common *J* for all other signals together with a value of *A*_*j*_*/h* within the expected range of 1–3 MHz also places the prediction of peak C in the correct chemical shift range. This argues for the apparent steeper temperature dependence of this signal to be of different origin, such as a temperature dependent conformational change [39].

A *J* value of 115 cm^−1^ is also comparable with that reported for spinach and algal ferredoxins (100 cm^−1^ and 115 cm^−1^) [35, 36]. The decrease in *J* value from oxidized to reduced state has been proposed to be the result of the larger ionic radius of Fe(II), which leads to a less efficient Heisenberg exchange mechanism between the two iron atoms [21]. Given that the *J* values for the oxidized and reduced states of FhuF fall within typical values for 2Fe–2S ferredoxins, and these values report on the coupling between the iron atoms via the inorganic sulfurs, these results strongly suggest that the core of the cluster is maintained in FhuF [40]. Mössbauer data show that only the parameters for the Fe(III) of the reduced cluster are unusual, whereas the parameters for the Fe(II) are typical for tetrahedral coordination. This is in agreement with paramagnetic ^1^H NMR experiments. This observation and the fact that the differences in the cluster binding motif sequence of FhuF vs the typical binding sequence for other ferredoxins is restricted to the separation of the first two cysteines in the sequence (C–C–*x*_10_–C–*x*_2_–C in FhuF vs the typical C–*x*_2_–C–*x*_8-15_–C–*x*_2_–C), we tentatively propose that the Fe(III) in the reduced state is bound to cysteines 244 and 245. Binding of vicinal cysteines to iron has precedent in the literature and was reported to lead to a cysS-Fe-Scys angle that is systematically wider than the ideal tetrahedral geometry, in agreement with the Mössbauer data for FhuF [11, 41]. In the present case it appears to lead also to different bonding strength by the two cysteines binding the Fe(III) in the cluster.

### FhuF binds ferrichrome and apo-ferrichrome

**Fig. 6:**
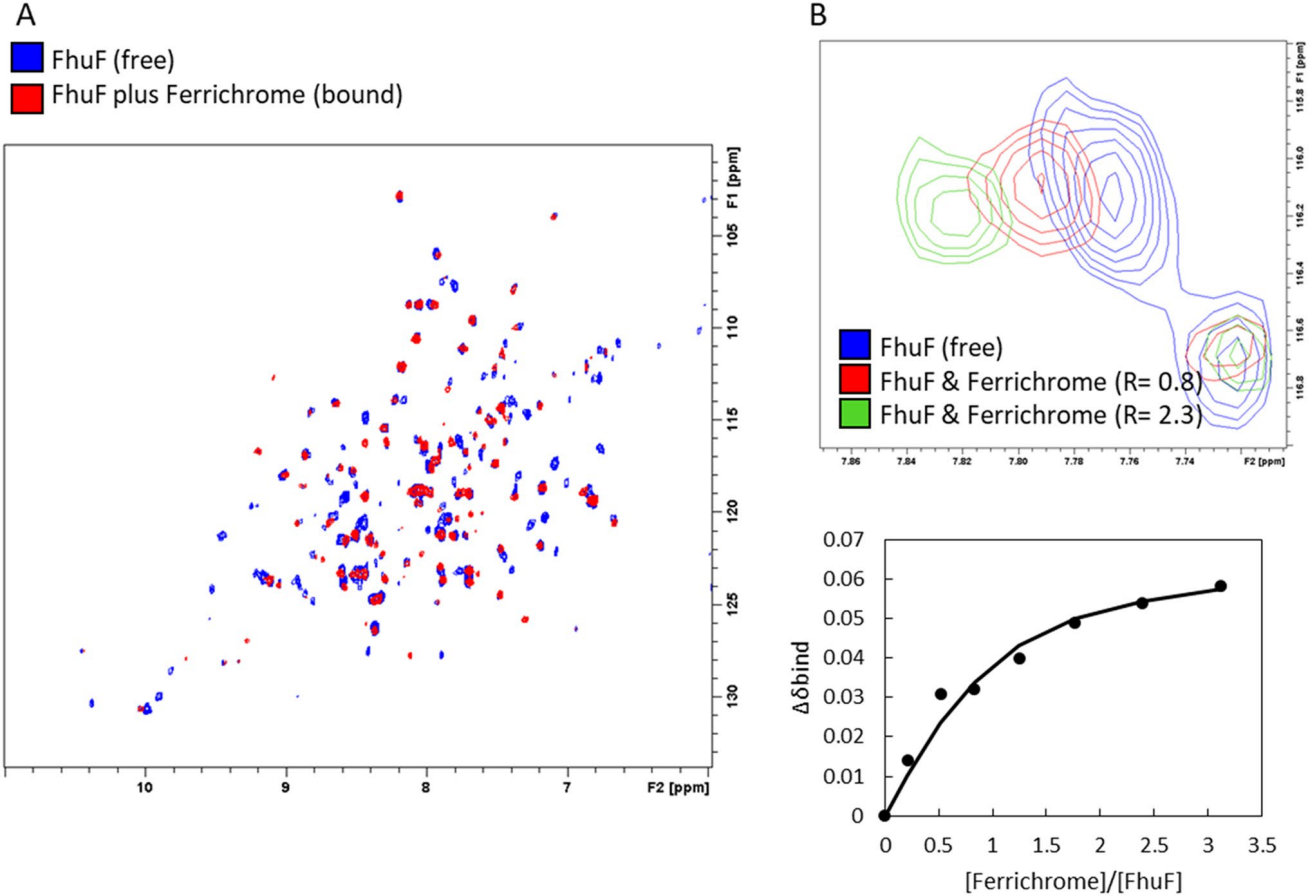
FhuF binds ferrichrome. 2D ^1^H^15^N TROSY-HSQC spectra of ^15^N^13^C-labeled FhuF portraying the spectral changes observed upon the addition of ferrichrome (**a**) and representative peak in fast-exchange regime with respective binding and fitting curve (**b**). R refers to the concentration ratio between ligand and protein

When oxidized, 2Fe–2S proteins are S’ = 0; however, some paramagnetism arises from the population of the excited states at room temperature. In FhuF, the effects of the 2Fe-2S paramagnetism are clearly reflected in the ^1^H^15^N TROSY-HSQC of FhuF, where only 215 backbone peaks out of 235 expected are observed (Figs. 1a, 6a). At least 20 peaks are undetected, and these most likely correspond to the residues that fall in the “blind sphere”, the region that surrounds the paramagnetic 2Fe–2S cluster [42]. Even with the use of paramagnetic-tailored experiments it was not possible to detect all the expected resonances [43, 44]. Nonetheless, upon the addition of ferrichrome to FhuF, spectral changes were observed (Fig. 6a), including the disappearance of peaks. These results are consistent with the binding of the Fe(III)-containing siderophore, which being also paramagnetic, leads to fast relaxation and peak broadening beyond detection in its vicinity [45]. Additionally, other spectral changes are observed, of which, peak shifts in the fast-exchange regime allow the determination of a dissociation constant of 53 ± 26 μM (Fig. 6b). Similarly, upon the addition of apo-ferrichrome, spectral changes also occur, suggesting the binding of apo-ferrichrome to FhuF. Surprisingly, as previously found with ferrichrome but to a lesser extent, some peaks are also bleached suggesting that not only the presence of the paramagnetic center (Fe(III) of ferrichrome) is contributing to this phenomenon (Figure S2A). Given the expected presence of cavities in the structure (Fig. 2a), it is likely that the binding of both apo- and holo-ferrichrome lead to conformational changes that bring further regions of the protein into the reach of the “blind sphere” generated by the 2Fe2S cluster.

### The reduction potential of FhuF is pH dependent broadening its catalytic capability

**Fig. 7:**
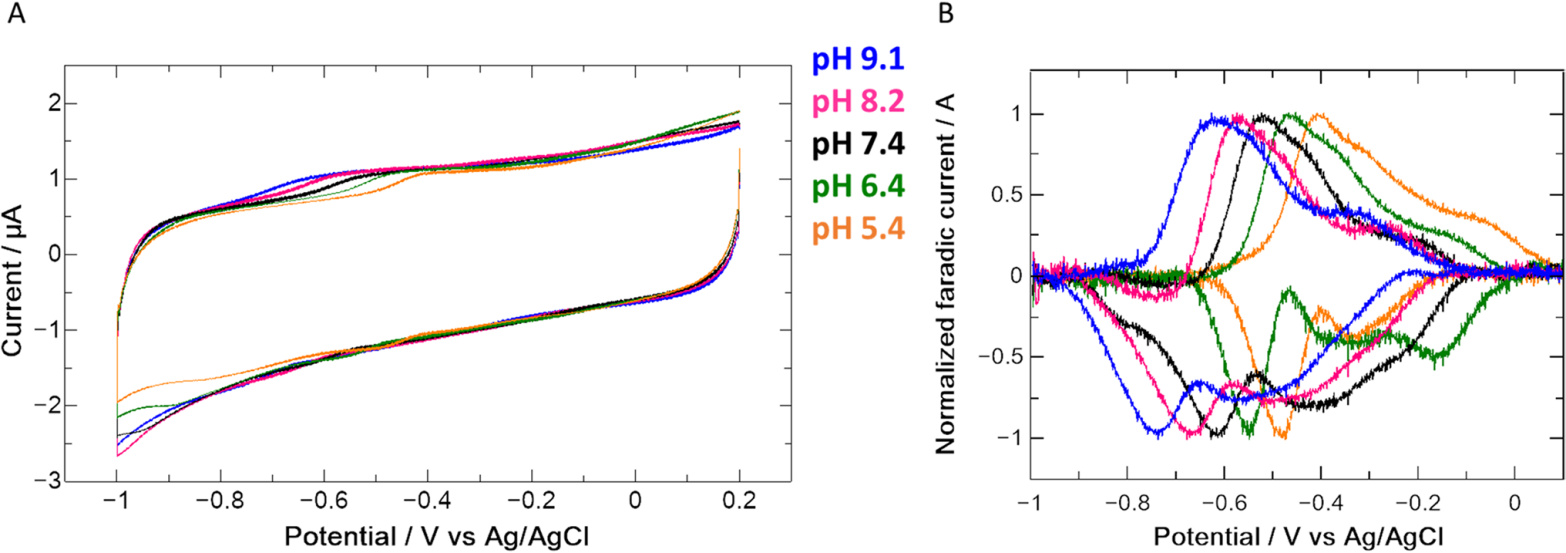
FhuF displays pH dependent redox properties. Raw voltammograms of FhuF adsorbed onto PGE recorded at 100 mV/s in 20 mM potassium phosphate buffer at pH 9.1 (blue), pH 8.2 (pink), pH 7.4 (black), pH 6.4 (green) and pH 5.4 (orange) **(a)** and Faradaic signal of FhuF obtained by subtracting the capacitive current from the raw voltammograms using QSoas software **(b)** [47]

FhuF does not display favorable electrochemical characteristics requiring extensive stabilization to observe a redox signal from the adsorbed protein onto PGE (Fig. 7a and Figure S3). This likely causes degradation of some of the protein at the surface of the electrode giving rise to the broad signal observed at higher potential in Fig. 7b, which is not reproducible in terms of potential or linewidth across different runs. By contrast, the signals at lower potential are reproducible and display a half-height width of the anodic and cathodic signals close to the theoretical value of 90 mV for a single electron transfer step (Fig. 7b) [46, 47]. The midpoint potential of – 370 mV vs SHE at pH 7.4 is consistent with earlier studies which report a reduction formal potential of − 310 ± 25 mV vs NHE at pH 7.3 for FhuF at cryogenic temperatures [8]. Cyclic voltammetry experiments performed at different pH values (Table 2) show that FhuF presents a redox-Bohr effect as also observed for a ferric-siderophore reductase from the SIP subfamily from *Shewanella frigidimarina* [12].

**Table 2:** Reduction potentials of FhuF as a function of pH determined from cyclic voltammetry measurements

pH	Formal potential (*V* vs SHE)
5.4	− 0.24 ± 0.07
6.4	− 0.30 ± 0.08
7.4	− 0.36 ± 0.09
8.2	− 0.42 ± 0.09
9.1	− 0.48 ± 0.09

The formal reduction potential changes by 60 mV for every pH unit in good agreement with the expectation for a Nernstian equilibrium for a coupled one proton and one electron transfer (2.3RT/nF). Fitting of the pH dependence of the potentials shows that the pka_ox_ value must be lower than 5 and the pka_red_ must be higher than 9 [48]. This is especially significant, since the normal range of *E.coli* growth can span pH 5 to 9 with some *E.coli* strains even surviving in lower acidic environments [49]. Therefore, the cyclic voltammetry data show that within this pH range the FhuF formal reduction potential shifts from − 250 mV to − 490 mV vs SHE. The lower value is particularly significant, since it broadens the diversity of siderophores that can be reduced by FhuF and provides a rational for the unexpected observation of Ferrioxamine B reduction reported in the literature [8].

## Conclusion

The FSR subclass of siderophore interacting proteins has thus far resisted characterization. In this work we obtained a structural and functional characterization of this important class of enzymes for iron uptake. FhuF binds both ferrichrome and apo-ferrichrome, advocating for its role as a bona-fide ferric siderophore reductase. The combination of SAXS data with the Rosetta derived model revealed that FhuF shares the “palm domain” with the siderophore biosynthetic enzymes, suggesting a common evolutionary origin that is distinct from that of SIP subfamily. SIPs, despite performing the same function, display significant structural homology with the diverse family of FAD/NAD(P)H oxidoreductases [50]. Paramagnetic NMR spectroscopy established that the perturbation of the cluster vs typical 2Fe–2S ferredoxins appears to be confined to the periphery involving only the cysteine ligands, in particular those coordinating Fe(III) in the reduced state showing the hallmarks of very different bond strengths for the two cysteines coordinating this iron. This asymmetric bonding of one cysteine to Fe(III) may be at the origin of the two conformations that appear to exist in the reduced state. Nonetheless, the information that the 2Fe–2S rhomboid core is not disturbed combined with the Mössbauer data and crystallographic knowledge of the geometrical consequences of vicinal cysteine binding to iron allowed us to tentatively assign the ligands of the two irons of the cluster. Knowing which of the two irons is redox active in the cluster is essential to understand the molecular mechanism of siderophore reduction by this protein once a more detailed structure is available. Interestingly, the presence of a redox-Bohr effect in FhuF shows that this aspect of molecular mechanism is common in siderophore-interacting proteins in both SIP and the FSR subfamilies. For the proteins in the SIP subfamily, that use a flavin co-factor, this is not surprising given the direct thermodynamic coupling of electron and proton transfer in the isoalloxazine ring. By contrast for proteins in the FSR subfamily it means that an acid–base residue that titrates in the physiological pH range needs to be placed near the 2Fe–2S cluster, eventually engaged in H-bonding interaction, to allow proton-electron coupling [51]. This strongly suggests that the pH dependence of the reduction potential in siderophore interacting proteins is a consequence of a common selective pressure on these proteins to enhance their physiological activity. Indeed, the broader redox range afforded by the pH dependence of the potential increases the diversity of siderophores that are accessible for iron extraction by this protein and provides a rational for the observation of reaction of FhuF with the low potential siderophore ferrioxamine B [8]. The present work sets the stage for a detailed investigation regarding the mechanism of ferric-siderophore reduction together with a detailed molecular characterization of the enzymes responsible for this process.

## Supplementary Information

Below is the link to the electronic supplementary material.

Supplementary file1 (PDF 224 KB)

## Abbreviations

ABC: ATP-binding cassette
CD: Circular dichroism
EPR: Electron paramagnetic resonance
FSR: Ferric-siderophore reductase
NMR: Nuclear magnetic resonance
SAXS: Small-angle X-ray scattering
SEC: Size exclusion column
SIP: Siderophore-interacting protein
TROSY-HSQC: Transverse relaxation optimized spectroscopy-heteronuclear single quantum coherence spectroscopy
