# Supplementary material for: Conjuring up a ghost: structural and functional characterization of FhuF, a ferric siderophore reductase from E. coli
Source: J Biol Inorg Chem. 2021 Feb 9;26(2):313–26. doi: 10.1007/s00775-021-01854-y (PMC8068687; doi:10.1007/s00775-021-01854-y)
Supplement: Supplementary file 1 — Supplementary file1 (PDF 224 KB) [file 775_2021_1854_MOESM1_ESM.pdf]

Supplementary information

Supp. Table

Table SI: SEC-SAXS data collection conditions

	**FhuF**
**Data acquisition**	
Beamline – Facility	B21-DSL
Wavelength (Å)	0.91
Sample-to-detector distance (m)	2.7
s range ($Å^{-1}$)	0.0036-0.4396
Concentration ($mg \cdot mL^{-1}$)	~9
HPLC system / SEC column	Agilent 1200 HPLC / Shodex KW402.5-4F
Detector	Pilatus 2M
Temperature (K)	283.15

Supp. Figures

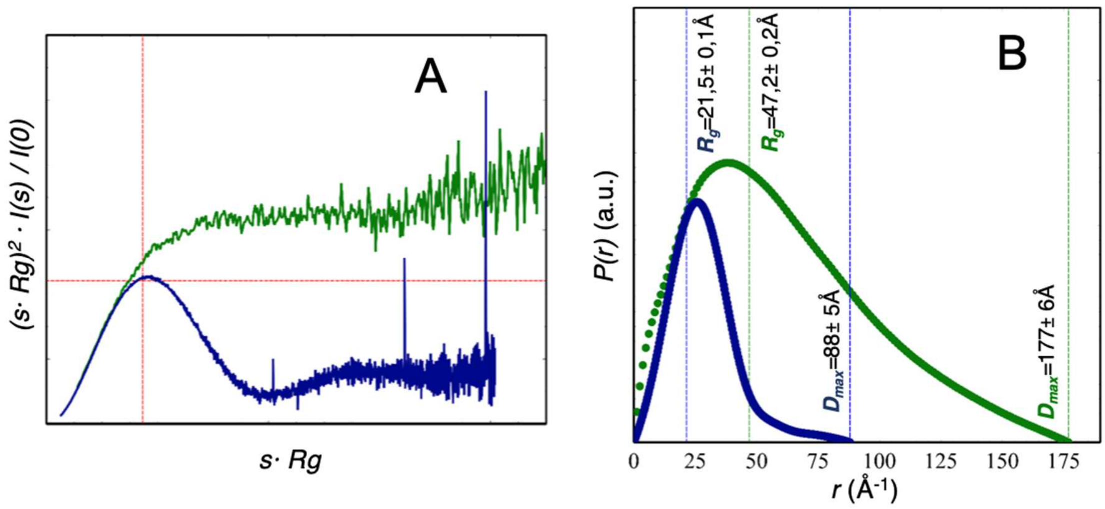

Figure S1. **Comparative SAXS: FhuF versus an IDP-like protein of 29 kDa. A**) Kratky representations of the SAXS patterns of FhuF (blue) and N-CoR-NID (dark green) next to their respective *P(r)* versus r profiles (**B**), plotted using the same color code. Dashed lines indicate the *Rg* and *Dmax* values. The SAXS experimental set for the region spanning from residue Gln2059 to Glu2325 of the Nuclear Receptor Co-Repressor (N-CoR-NID) was obtained from the curated repository for scattering data SASDB (www.sasbdb.org), with the entry code SASDF34. N-CoR-NID is a monomeric intrinsically disordered protein with a molecular mass of 29 kDa, similar to the molecular weight of FhuFΔ1-17 (28kDa). Contrary to N-CoR-NID, the Kratky plot of FhuF has a clear peak maximum around $sRg = \sqrt{3}$ (red dashed line), reflecting globularity. Besides the similar mass, the disordered N-CoR-NID has a larger D_{max} and smoother ending to $P(D_{max})=0$ than the globular FhuF.

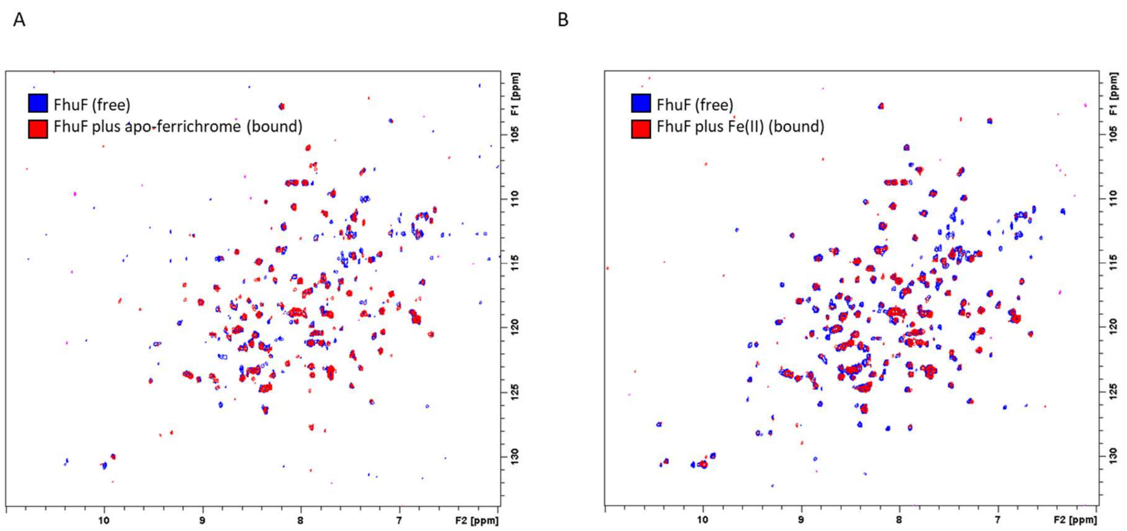

Figure S2 - 2D $^{1}H^{15}N$ TROSY-HSQC spectra of ^{15}N13C-labeled FhuF portraying the spectral changes observed upon the addition of apo-ferrichrome **(A)** 2D $^{1}H^{15}N$ TROSY-HSQC spectra of $^{15}N^{13}C$-labeled FhuF portraying the spectral changes observed upon the addition of Fe (II) **(B)**.

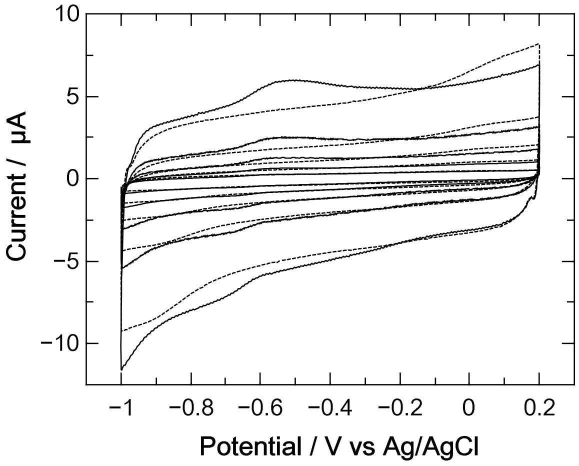

Figure S3 - Raw voltammograms of unmodified (dashed line) and FhuF-modified (solid line) PGE recorded at different scan rates (20 ; 50 ; 100 ; 200 ; 500 mV/s) in 20 mM potassium phosphate buffer pH 7.9.
